# Identification of new reference genes for the normalisation of canine osteoarthritic joint tissue transcripts from microarray data

**DOI:** 10.1186/1471-2199-8-62

**Published:** 2007-07-25

**Authors:** Lindsey J Maccoux, Dylan N Clements, Fiona Salway, Philip JR Day

**Affiliations:** 1 Centre for Integrated Genomic Medical Research, University of Manchester, The Stopford Building, Oxford Rd, M13 9PT Manchester, UK; 2 The Musculoskeletal Research Group, Faculty of Veterinary Science, University of Liverpool, L69 3BX Liverpool, UK; 3 ISAS – Institute for Analytical Sciences, Bunsen-Kirchhoff-Str. 11-13, D-44139 Dortmund, Germany

## Abstract

**Background:**

Real-time reverse transcriptase quantitative polymerase chain reaction (real-time RT-qPCR) is the most accurate measure of gene expression in biological systems. The comparison of different samples requires the transformation of data through a process called normalisation. Reference or housekeeping genes are candidate genes which are selected on the basis of constitutive expression across samples, and allow the quantification of changes in gene expression. At present, no reference gene has been identified for any organism which is universally optimal for use across different tissue types or disease situations. We used microarray data to identify new reference genes generated from total RNA isolated from normal and osteoarthritic canine articular tissues (bone, ligament, cartilage, synovium and fat). RT-qPCR assays were designed and applied to each different articular tissue. Reference gene expression stability and ranking was compared using three different mathematical algorithms.

**Results:**

Twelve new potential reference genes were identified from microarray data. One gene (mitochondrial ribosomal protein S7 [*MRPS7*]) was stably expressed in all five of the articular tissues evaluated. One gene HIRA interacting protein 5 isoform 2 [*HIRP5*]) was stably expressed in four of the tissues evaluated.  A commonly used reference gene glyceraldehyde-3-phosphate dehydrogenase (*GAPDH*) was not stably expressed in any of the tissues evaluated. Most consistent agreement between rank ordering of reference genes was observed between *Bestkeeper©* and geNorm, although each method tended to agree on the identity of the most stably expressed genes and the least stably expressed genes for each tissue. New reference genes identified using microarray data normalised in a conventional manner were more stable than those identified by microarray data normalised by using a real-time RT-qPCR methodology.

**Conclusion:**

Microarray data normalised by a conventional manner can be filtered using a simple stepwise procedure to identify new reference genes, some of which will demonstrate good measures of stability. Mitochondrial ribosomal protein S7 is a new reference gene worthy of investigation in other canine tissues and diseases. Different methods of reference gene stability assessment will generally agree on the most and least stably expressed genes, when co-regulation is not present.

## Background

The quantification of gene expression allows the mechanism organising biological activity to be determined. At present, real-time RT-qPCR provides the most accurate and specific measure of gene expression, with an unsurpassed dynamic range and a high level of reproducibility.

A number of variables will contribute to the variability of gene expression measurements, such as the number and type of cells in the tissue evaluated, the method and efficiency of mRNA extraction, mRNA handling techniques [1], mRNA integrity [2,3], method of reverse transcription [4] and analytical detection chemistry method [1]. These inter-sample differences are addressed through the process of normalisation [5], whereby the expression of an individual gene within a sample is related to that of a calibrating gene known as a reference, control or "housekeeping" gene. Ideally, a reference gene is expressed at a consistent and repeatable quantity across all samples being compared, so that relative differences in gene expression can be measured with confidence. Commonly used reference genes such as beta-2-microglobulin (*B2M*), glyceraldehyde-3-phosphate dehydrogenase (*GAPDH*) and beta actin (*ACTB*), are not constantly expressed across all tissue types and disease states [6,7]. Thus it is widely accepted that the selection of reference genes should be established through the validation of expression stability in the tissue or cells of interest, before use.

A number of statistical algorithms exist for the optimisation of reference gene selection, such as geNorm [6], Global Pattern Recognition [8], *Bestkeeper©*[9], equivalence tests [10] and NormFinder [11]. In each case, mathematical evaluation of expression data allows the ordering of candidate reference genes, on the basis of the relative expression stabilities. At present, no gold standard exists for the selection of reference genes, and although methods have been compared with similar results in some reports [12-14] but not in others [11], the optimal method for selections remains unknown.

The identification of new reference genes from microarray data, within a particular tissue type, has been demonstrated to provide more "stable" reference genes than those conventionally used [11,14-16], as determined using stability algorithms. Microarray data can be stratified on the basis of fold changes in expression [14], the variance of expression [11,16] or integrative correlations [15]. Candidate genes can then be selected from stratified data, and frequently demonstrate expression stabilities greater than conventionally used reference genes [11,14,15]. However microarray data has yet to identify a new reference gene which shows consistent stability across multiple tissue or cell types, and/or disease situations. Therefore a ubiquitous reference gene suitable for normalisation of gene expression of all experiments probably does not exist, but the identification of new reference genes to improve in reference gene stability is important to reduce error in RT-qPCR experiments.

In this study, we identified candidate reference genes from microarray expression profiling data generated from the evaluation of two different canine articular tissues (cartilage and cranial cruciate ligament). The relative stability of expression of each reference gene in normal and osteoarthritic canine articular tissues was determined from RT-qPCR reactions using statistical algorithmic packages. The stability of the new reference genes were compared between tissues, and related to a commonly used reference gene(*GAPDH*).

## Results

### New reference genes

Identities and putative functions of each of the new reference genes are listed in Table 1. Although the genes selected did not localise to common pathways or functions, two of the genes coded for mitochondrial ribosomal proteins. The metrics of the candidate reference gene stability are presented in Table 2.

**Table 1:** A list of the gene annotations, functions, primer and probe sequences, and qPCR metrics for the 12 new reference genes, and glyceraldehyde 3-phosphate dehydrogenase

**Gene Name**	**Gene Function**	**Gene Symbol**	**Accession Number [GenBank]**	**Forward(F) and Reverse(R) Primers**	**Probe Sequence**	**Average Standard Deviation of Triplicate**	**R**^2^	**PCR Efficiency**
CG14980-PB	Protein coding	*C7orf28B*	XM_536878	F-gcaggaagggattctccag R-ggtccagtaagaaatcttccataa	gccaggaa	19.8	0.986	104.3
Glyceraldehyde-3-phosphate dehydrogenase	Enzyme in the glycolysis/gluconeogenesis pathway	*GAPDH*	NM_001003142	F-ctggggctcacttgaaagg R-caaacatgggggcatcag	ctgctcct	20.3	0.991	101.1
Gu binding protein	Nuclear receptor in transcriptional co-regulation	*PIAS1*	XM_535524	F-ggagacaatcagcattataacacct R-tgatcatctgacactgctgct	ggctgctg	16.9	0.990	99.6
HIRA interacting protein 5 isoform 2	Histone-interaction-DNA packaging	*HIRP5*	XM_850340	F-aattcagaacatgctgcaatttta R-tgattcatcatccataacctgttc	aggtggag	8.6	0.998	96.9
Hematopoietic stem/progenitor cells 176	Transport protein particle involved in endoplasmic reticulum to Golgi vesicle transport	*TRAPPC2L*	XM_844929	F-gatgatccaggtgtgctgag R-caatacggttatgtcaacagcact	ctggagga	25.2	0.993	97.2
5-aminoimidazole-4-carboxamide ribonucleotide formyltransferase/IMP cyclohydrolase	Purine biosynthesis	*ATIC*	XM_858011	F-cgctgcctctttcaaacat R-tttggcctcatcttcactgag	cagcaggt	13.4	0.991	97.7
Mitogen-activated protein kinase 6	Phosphorylates microtubule-associated protein 2 (MAP2)	*MAPK6*	XM_858091	F-tcttcttgggatagccagtttg R-cctcacctcacaacaaaactgat	ggtggtgg	14.9	0.992	97.6
Mitochondrial 28S ribosomal protein S25	Mitochondrial ribosomal subunit protein synthesis	*MRPS25*	XM_533729	F-tgaaggtcatgacggtgaac R-tggatctgaggtatgttgaaaaac	gccaggaa	14.6	1.000	95.5
Cytoplasmic protein NCK2	Regulates cell proliferation	*NCK2*	XM_538440	F-cagacgctctacccgttca R-gtctcgcccttctcgaagtt	aggaggag	28.7	0.975	96.7
ORM1-like 2	Protein folding in the endoplasmic reticulum	*ORMDL2*	XM_843143	F-atggactacgggctccaat R-ctggccaggaggtagagtaca	ctcctccc	28.2	0.996	103.1
Phosphatidylserine synthase I	Membrane bound protein that catalyses the replacement of phospholipids by L-serine	*PTDSS1*	XM_849686	F-actcagaatgcgacgatgg R-tcagaaccttttgaacctttcg	ctggtctc	15.3	0.996	100.9
Mitochondrial ribosomal protein S7	Mitochondrial protein synthesis	*MRPS7*	XM_846915	F-agtgcagggagaagaagcac R-cagcagctcgtgtgacaact	ggatgctg	12.1	0.998	100.8
Transketolase	Enzyme in pentose phosphate pathway	*TKT*	XM_533792	F-caacttctgtggctcccact R-ccagatcttccagagccatc	tggggaag	11.8	0.993	103.4

**Table 2:** Correlation coefficients for the rank ordering of gene stability by different reference gene analysis methods

**Tissue**	**Method**	**NormFinder**	**GeNorm**
**Cartilage**	**GeNorm**	0.462	
	**BestKeeper**	0.515	0.721
**Cruciate**	**GeNorm**	0.835	
	**BestKeeper**	0.915	0.794
**Synovium**	**GeNorm**	0.833	
	**BestKeeper**	0.745	0.579
**Fat Pad**	**GeNorm**	0.907	
	**BestKeeper**	0.867	0.939
**Bone**	**GeNorm**	0.710	
	**BestKeeper**	0.382	0.475

### Articular cartilage

**Figure 2:**
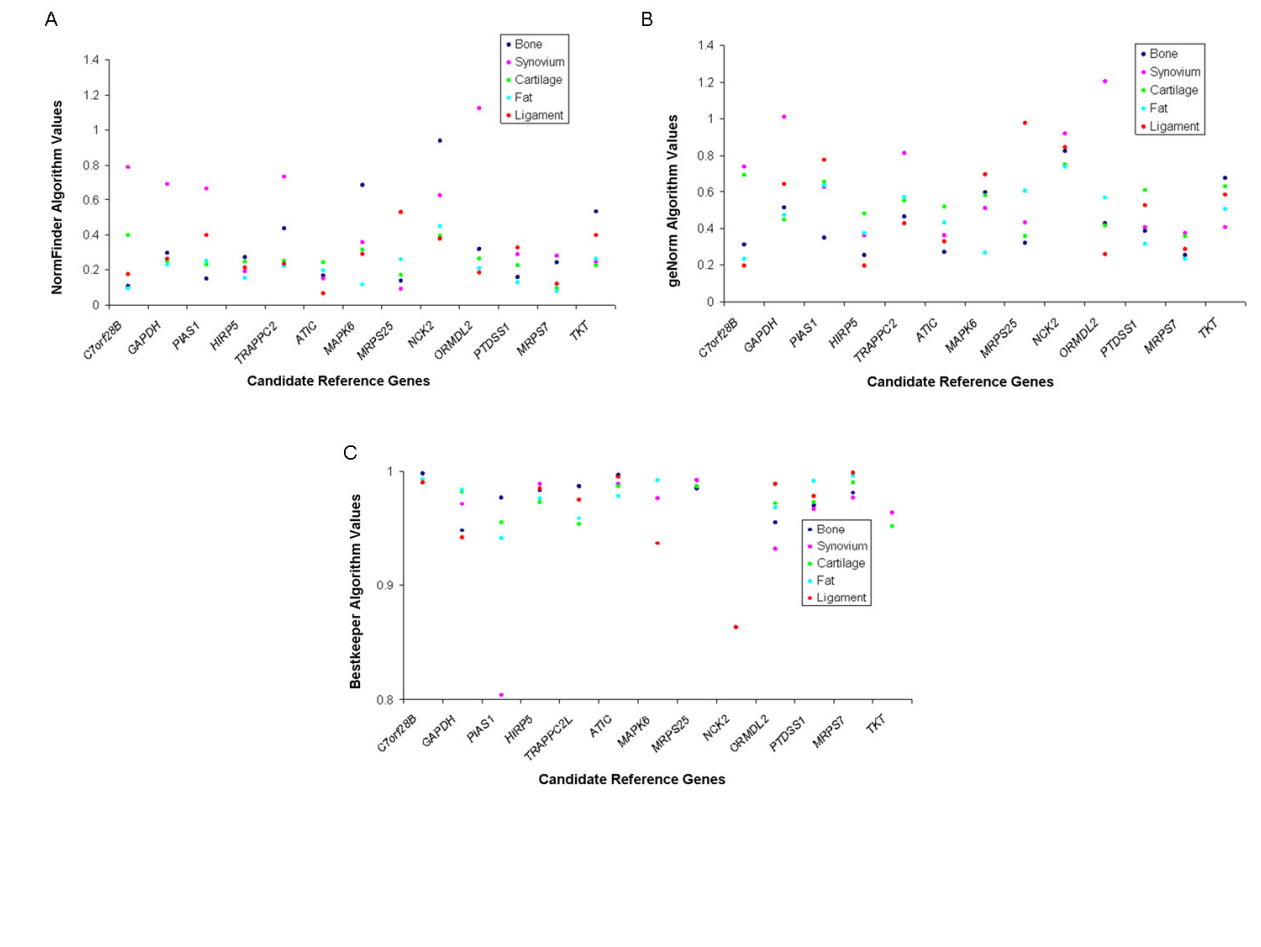
Reference gene stability measures as determined by: 2A. The NormFinder Algorithm (with a lower value indicating increased reference gene stability). 2B. The geNorm algorithm (with a stability measure [M value] <0.4 indication appropriate reference gene stability). 2C. The Bestkeeper algorithm (with a higher value indicating increased reference gene stability). Please note that as only the top 10 genes (as ranked by the NormFinder algorithm) are selected for analysis, thus there are not necessarily data points for each gene corresponding to each tissue.

All methods of stability analysis agreed in finding the new genes *MRPS7 *and *MRPS25 *to be stably expressed. Likewise, *C7orf28B *and *NCK2 *were determined to be the least stably expressed genes by both geNorm (Figure 2B) and NormFinder (Figure 2A). *GAPDH *was identified as the 4^th ^most stably expressed gene by both geNorm and *Bestkeeper©*, and the 8^th ^most stably expressed gene by NormFinder.

### Infrapatella fat pad

All three methods of reference gene analysis agreed on the most stably expressed reference genes, which were *C7orf28B*, *MRPS7 *and *MAPK6*. GeNorm (Figure 2B) and NormFinder (Figure 2A) agreed that the least stably expressed gene was *NCK2*. *GAPDH *was identified as the 9^th ^most stably expressed gene by NormFinder, the 7^th ^most stably expressed gene by geNorm, and the 5^th ^most stably expressed gene by *Bestkeeper©.*

### Cranial cruciate ligament

Methods did not agree on the most stably expressed genes, although all methods agreed on the five most stably expressed genes (albeit, not their order); *ATIC*, *MRPS7*, *C7orf28B*, *ORMDL2 *and *HIRP5*. *MRPS25 *was the least stably expressed gene as determined by both NormFinder (Figure 2A) and geNorm (Figure 2B). *GAPDH *was identified as the 7^th ^most stably expressed gene by NormFinder, the 9^th ^most stably expressed gene by geNorm, and the 8^th ^most stably expressed gene by *Bestkeeper©.*

### Synovial membrane

Although *Bestkeeper©* and NormFinder agreed on the six most stably expressed genes (*MRPS25*, *ATIC*, *HIRP5*, *TKT*, *MRPS7*, *PTDSS1*), and *NCK2 *was determined to be the least stably expressed gene by NormFinder (Figure 2A) and geNorm (Figure 2B), no further patterns of agreement in rank ordering of the expression profiles were identified. *ATIC *was identified as the most stably expressed gene by NormFinder (Figure 2A) and *Bestkeeper©* (Figure 2C), and the 6^th ^most stably expressed gene by geNorm.

### Bone

Rank ordering between NormFinder and geNorm agreed on the seven most stably expressed (*C7orf28B*, *MRPS25*, *PIAS1*, *PTDSS1*, *ATIC*, *MRPS7 *and *HIRP5*) genes but not their order, and the least stably expressed gene (*NCK2*). *Bestkeeper©* (Figure 2C) and NormFinder (Figure 2A) agreed on the most stably expressed gene (*C7orf28B*).

### Comparison of reference gene performance in all tissues

Using the reference gene stability value (M) of 0.40 as the determinant of stable expression [6], *MRPS7 *was stably expressed in all five tissues, and *HIRP5 *was found to be stably expressed in four tissues (Figure 2B). *GAPDH *was found to be unstable in all of the tissues evaluated, which is consistent with the findings of a previous study of reference genes in these tissues [17]. Comparison of gene stability (M) and pairwise stability (V) values with a previous study of commonly used reference genes using similar tissues further illustrates how optimal reference gene stabilities, as assessed by geNorm, can be achieved using the new reference genes rather than the commonly used reference genes (Table 3).

**Table 3:** Comparison of M and V values generated in this study when compared to a previous study evaluating similar tissues

	**Current Study**			**Ayers (2006) Study [17]**		
**Tissues**	**Reference Genes**	**M (Gene Stability) Value**	**V (Pairwise Stability) Value**	**Reference Genes**	**M (Gene Stability) Value**	**V (Pairwise Stability) Value**

**Articular**	*MRPS7*	0.37	0.122	*RPL13A*	0.57	0.31
**Cartilage**	*MRPS25*			*SDHA*		
**Synovium**	*MRPS7*	0.2	0.091	*N/A*	N/A	N/A
	*ATIC*					
**Cruciate**	*HIRP5*	0.2	0.093	*B2M*	0.59	0.27
**Ligament**	*C7orf28B*			*TBP*		
**Fad Pad**	*C7orf28B*	0.23	0.088	*B2M*	1.02	0.35
	*MRPS7*			*SDHA*		
**Bone**	*MRPS7*	0.36	0.084	*N/A*	N/A	N/A
	*HIRP5*					

No single reference gene was consistently identified as being the most stably expressed by NormFinder, geNorm or *Bestkeeper©* across most tissues. There was not consistent agreement in the rank ordering, or the selection of the optimal candidates by the different methods, although agreement was generally reached on the most and least stable gene. For example *Bestkeeper©* and NormFinder always identified the same gene as being most stably expressed. When looking at rank order across all three reference gene stability programs, fat pad showed the highest correlation between methods, followed by cruciate ligament, cartilage, bone and synovium as the least consistent (Table 2).

When the data for all tissues was compared together (Figure 2A, B, C), a much clearer pattern of reference gene stability was observed. The stability metrics of the reference genes in different tissues show similar patterns across all three methods. *MRPS7 *demonstrates the most consistent metric (low geNorm M value, low NormFinder value and high *Bestkeeper©* correlation), with *HIRP5 *and *ATIC *demonstrating a similarly consistent stability across all tissues. This is supported by the finding that *MRPS7 *was consistently identified as being stably expressed in all tissues by geNorm (*MRPS7*), as well as being ranked as one of the two most stable reference genes in four of the five tissues by geNorm (cartilage, fat, bone and synovium), and in three of the five tissues using NormFinder and *Bestkeeper©* (cartilage, ligament and fat).

### Comparison genes identified by different methods

Identification of new reference genes using RT-qPCR methodology for gene normalisation was not successful at identifying new reference genes with increased stability. *NCK2 *was determined to be the least stably expressed gene in synovium and fat pad, and one of the four least stably expressed genes in cruciate ligament and cartilage. *TRAPPC2L *was not identified as being stably expressed in any tissue using the geNorm algorithm, and was not ranked higher than the 8^th ^most stably expressed gene in any tissue using the NormFinder algorithm.

## Discussion

A number of different strategies have been employed to filter microarray data to identify new reference genes, such as selection on the co-efficient variation and level of expression [11], fold changes of expression [14,16], or integrative correlations [15]. We used a combination of filtering on statistical significance, fold change and coefficient of variation (percentage standard deviation) to narrow the potential number of reference genes. Furthermore, these criteria were applied to three different experiments, using two different data sets, to identify genes which were more likely to have generic stability across multiple tissues for diseases. Genes were finally filtered on the basis of defined annotation and level of expression. In retrospect, genes should also have been selected on the basis of single transcript expression (i.e. the absence of splice variants). Although the two most stably expressed genes (*MRPS7* and *HIRP5*) currently have no splice variants reported, the absence of splice variants did not necessarily confer reference gene stability across multiple tissues (as demonstrated by *GAPDH* and *MRPS25*, genes which do not have splice variants annotated and were not the most stably expressed) but should be taken into account when selecting new reference genes, as another potential indicator of instability. Our filtering method was straightforward, quickly performed and easily completed by any person without a full understanding of microarray data set handling, and as such could be applied to publicly available microarray data sets for a given experiment or disease.

Variability in the expression of commonly used reference genes has been recognised on the analysis of cell culture experiments [18] and clinical tissue specimens [19]. The selection of reference genes upon their stability as determined by the mathematical assessment of their expression values is a widely accepted technique [6,12-15,20,21]. We identified one gene which showed stable expression across normal and diseased articular tissues (*MRPS7*), and a number of genes which demonstrated a relatively consistent stability across the majority of tissue specimens (*HIRP5*). One should bear in mind that the tissues evaluated were from the same embryological origin (mesenchymal tissue), and hence there may have been a tendency towards identifying a reference gene which was stable in all tissues, although this is not supported by reports of reference gene stability in different tissues [21]. Likewise, the diseases compared in the microarray data sets were the same as those affecting the tissue samples evaluated by real-time RT-qPCR, which may further tend towards identifying reference genes whose stability was constant. Therefore, although we identified one gene as being stably expressed in all tissues, we would not advocate its use as a reference gene in other tissues or diseases without assessment of its stability in the samples to be evaluated [6,16,21], as the utopia of a universal reference gene suitable for all studies probably does not exist on basis of the published evidence to date.

Mitochondrial ribosomal protein S7 is involved in mitochondrial protein synthesis. The precise function of this gene is unknown in eukaryotes, but the protein is thought to be involved in organising the 3' domain of the 16 S rRNA in the mitochondria of prokaryotes, and thus be involved in the initiation of translation in mammalian mitochondria [22]. Microarray data analysis indicated the mitochondrial ribosomal protein S25 was also stably expressed, although it was only stably expressed in two of the four tissues analysed by RT-qPCR (cartilage and fat pad). In a separate study, mitochondrial ribosomal protein L19 was one of six genes identified from microarray data obtained from different tissues and cells, as a good reference gene for real-time RT-qPCR experiments, when compared to conventional reference genes for mammary tumour expression profiling [16]. Mitochondrial ribosomal gene expression appears to show greater stability across different tissues and thus may be less affected by tissue type or disease status, and better potential candidate reference genes for other real-time RT-qPCR experiments.

Comparing the results of this study to a similar previous study of commonly used reference genes in multiple articular tissues demonstrates the increased stability of the "new" reference genes (Table 3) [17]. The selection of candidate reference genes from microarray data identified new genes which were more stably expressed and is consistent with the general outcome of previous studies using this methodology [11,14-16]. The normalisation of microarray data by geometric mean of three reference genes [6] did not identify genes (*NCK2 *or *TRAPPC2L*) with appropriate stability to be suitable for use as reference genes. The instability of these genes may be reflected, in part, by the greater variation identified in the triplicate repeats of each assay when compared to more genes determined as being more stably expressed such as *HIRP5 *or *MRPS7*. The less stable expression of the three conventional reference genes (*GAPDH*, *RPL13A *and *SDHA*) probably resulted in the selection of similarly "unstably" expressed reference genes from microarray data, and thus accounted for this being a futile method of trying to select reference genes, which agrees with the evaluation of these types of methodologies for the accurate normalisation of microarray data [23]. These genes were selected on the basis of a preliminary study of reference gene stability in canine OA tissues [24], however subsequent work evaluating greater sample numbers has determined that one of these genes (*SHDA*) demonstrates differential expression in OA cartilage [25] and thus its use may have further predisposed to the selection of genes which were not stably expressed. Furthermore, the conventionally used reference gene we evaluated (*GAPDH*) did not show acceptable stable expression in any of the tissues we analysed.

We used three different methods of ranking reference gene stability in each experiment. Correlation co-efficient could be generated to compare methods and quantify the agreement of the rank ordering of different methods. Previous studies have demonstrated that the generation of rank orders can be very similar between different methods [14], but this is not always the case [11]. The best correlation in rank ordering was observed between geNorm and *Bestkeeper©*, across all the tissues which is unsurprising as both are generated by pairwise comparisons (although geNorm uses transformed data, whereas *Bestkeeper©* uses threshold cycle values), although *Bestkeeper©* and NormFinder always identified the same gene as being most stably expressed. The rank order of reference gene stability was identified most consistently for fat pad, followed by cruciate ligament, cartilage, bone and least consistently for synovium.

The advantage of using a model based stability assessment is that rank ordering can be changed if co-regulated genes are included in the stability assessment procedure, as pairwise assessments will determine an increase in stability between these methods [11]. As we identified a number of new reference genes which have very little functional information associated with their annotation, we checked for co-regulation between the most stably expressed genes by removing one of the highest ranked genes (as determined by pairwise comparisons) alternately, and re-assessing the rank ordering of reference genes stabilities. No major changes in rank ordering or reference gene stability were observed when this was performed. However, it should be noted that other factors besides gene expression pathway similarities can contribute to co-regulation. Yu et al. (2003) identified that genes targeted by similar transcription factors have complex relationships across the co-regulated genes [26].  The different methods for determining reference gene stability did not necessarily agree on rank order, but were good at determining both the most and least stably expressed genes, regardless of method. The top two most stably expressed genes analysed by geNorm for each tissue were then used to study cytokine gene expression in canine osteoarthritis [27].

## Conclusion

The use of microarray data for the selection of reference genes allowed the identification of multiple genes demonstrating greater stability than a conventional reference gene in multiple tissues. Mitochondrial ribosomal protein S7 is suitable for use in all the experimental conditions we analysed, and is suitable for investigation in other experiments. Different methods of assessment of gene stability do not always show correlation between the rank order of gene expression stability, but they do generally agree on which genes are suitable for use to normalise gene expression experiments.

## Methods

### Microarray data

Expression profiling data from 10 hip articular cartilage samples (5 control, 5 from osteoarthritic [OA] joints) and 16 cranial cruciate ligament (CCL) samples (4 normal low-risk of rupture, 7 normal high-risk of rupture, and 5 ruptured ligament from OA joints) were generated from a custom designed 44 k transcript canine whole genome 60 mer oligonucleotide microarray [28]. Raw data was normalised by two methods; locally weighted scatterplot smoothing (LOWESS), or using the geometric mean of 3 conventional reference genes arbitrarily selected (glyceraldehyde-3-phosphate dehydrogenase [*GAPDH*], ribosomal protein L13a [*RPL13A*], succinate dehydrogenase flavoprotein subunit A [*SDHA*]). Expression quantification was exported into an Excel Datasheet (Microsoft Excel 2003), and the data compared in three separate experiments as follows;

1) Normal hip articular cartilage was compared to OA cartilage,

2) Normal CCL (high-risk of rupture) was compared to normal CCL (low-risk of rupture),

3) Normal CCL (high risk of rupture) was compared to ruptured CCL

### Selection of reference gene candidates

**Figure 1:**
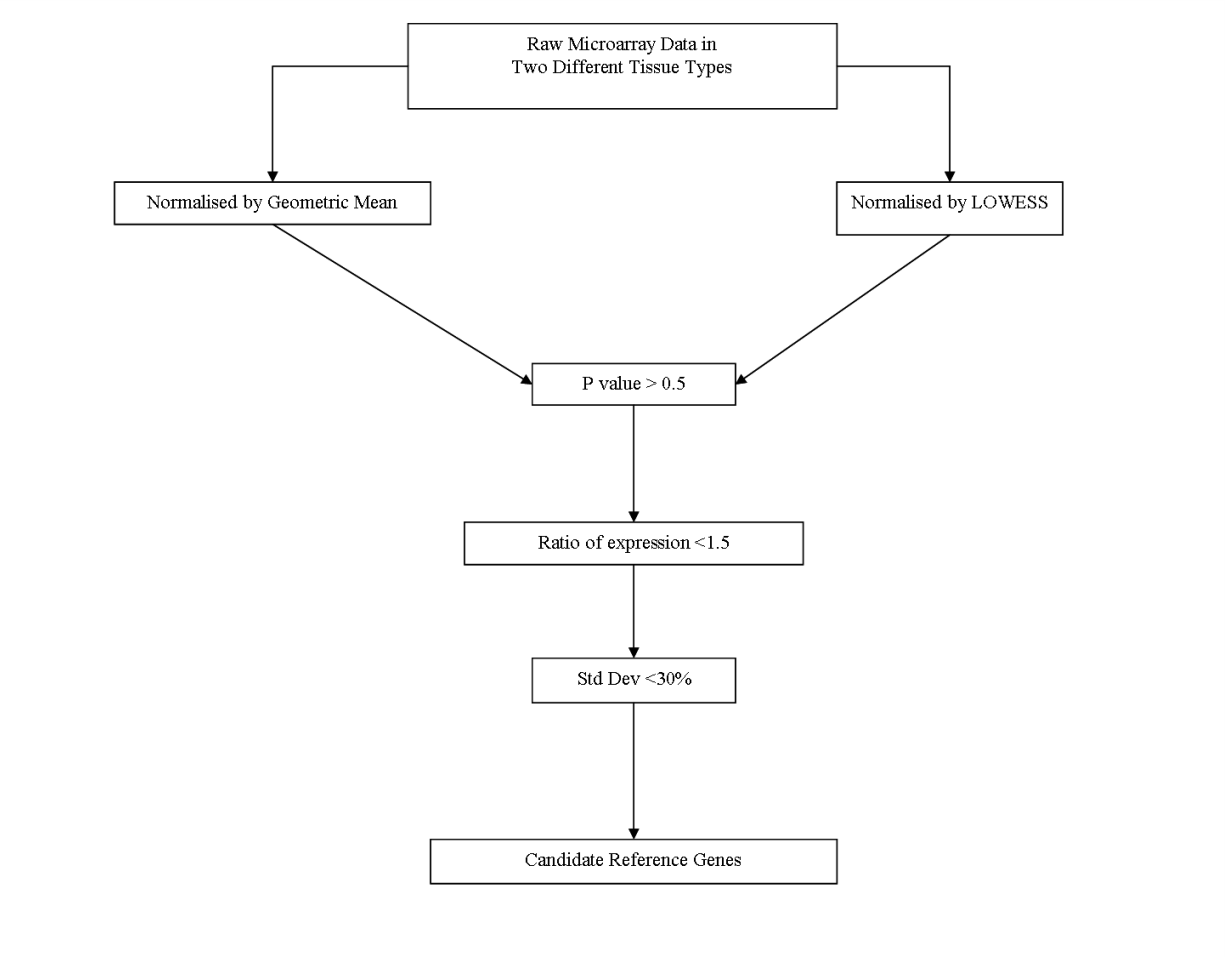
Microarray data normalised by two different methods was filtered to identify new reference genes using statistical significance, fold changes in expression between experimental groups, the co-efficient of variation and ontological evaluation

The stepwise procedure for identifying candidate reference genes is illustrated in Figure 1. Data for each reference gene candidate was compared in each experiment by calculating the fold change in mean expression level (between the two comparison groups), student's t-tests and percentage standard deviation (co-efficient of variation). To identify the most stably expressed genes across each of the experiments, the prospective reference genes were then selected using the following the criteria;

1. Student's t-test P value > 0.5 (in all experiments).

2. Ratio of expression between the two groups compared in each experiment <1.5 (in all experiments).

3. Standard deviation of the mean expression in each experimental group being <30% (in all experiments).

The data sets were reduced to 420 transcripts (LOWESS normalised) and 13 transcripts (reference gene normalisation). To further refine and filter the new reference gene list, data was ordered on the average signal intensity and;

4. The probe sequences used from the microarray experiments were entered into the NCBI BLAST^® ^database [29] to confirm the gene identity,

5. Gene function was determined [29] and the associated gene information checked to ensure no known involvement in OA.

Complete filtering reduced the data set to 12 genes, of which 10 were selected from the LOWESS normalised data, (CG14980-PB [*C7orf28B*], Gu binding protein [*PIAS1*], HIRA interacting protein 5 isoform 2 [*HIRP5*], 5-aminoimidazole-4-carboxamide ribonucleotide formyltransferase/IMP cyclohydrolase [*ATIC*], Mitogen-activated protein kinase 6 [*MAPK6*], Mitochondrial 28S ribosomal protein S25 [*MRPS25*], ORM1-like 2 [*ORMDL2*], Phosphatidylserine synthase 1 [*PTDSS1*], Mitochondrial ribosomal protein S7 [*MRPS7*] and Transketolase [*TKT*]), and 2 were selected from the reference gene normalised data (Hematopoietic stem/progenitor cells 176 [*TRAPPC2L*] and Cytoplasmic protein NCK2 [*NCK2*]). Glyceraldehyde-3-phosphate dehydrogenase [GAPDH] was also selected as it is a commonly used reference. The sequence details and putative functions (determined by reference to the human transcripts [29]) are listed in Table 1.  

### Sample collection and storage

A separate set of samples were collected for the analysis of the new reference genes. Infrapatellar fat (n = 5), ruptured cranial cruciate ligament (n = 5), femoral head articular cartilage (n = 5), ulnar subchondral bone (n = 5) and synovial membrane (n = 5) were obtained from dogs with clinical OA secondary to naturally occurring joint disease. In each case the samples were obtained as part of the standard surgical treatment for the disease in question (total hip replacement, cranial cruciate ligament rupture surgery or fragmented coronoid process removal). Control samples (healthy) were obtained from infrapatellar fat pad (n = 5), cranial cruciate ligament (n = 5), synovial membrane (n = 5), hip articular cartilage (n = 5) and ulnar bone (n = 5) of dogs euthanized for reasons other than, and with no evidence of, joint disease. Following the collection of the tissue, the samples were weighed and immediately stored in RNAlater™ (Qiagen Inc, Crawley, UK), according to the manufacturer's instructions, until extraction.

### RNA extraction

For all of the tissue samples total RNA was extracted using a phenol/guanidine hydrochloride reagent (Trizol, Invitrogen Ltd, UK) with a chloroform extraction and ethanol precipitation, as previously described [30]. An on column DNA digestion step was included (RNase-Free DNase Set, Qiagen Ltd, Crawley, UK). Final elution of the total RNA was performed using 30 μl of RNase free water, and repeated to maximize the amount of RNA eluted. Total RNA samples were stored at -80°C until use. The concentration of total RNA representing each sample was quantified by using a NanoDrop^® ^ND – 1000 UV/Visible Spectrophotometer (NanoDrop Technologies Ltd, Utah, USA).

### cDNA synthesis

Reverse transcription was performed using Superscript II reverse transcriptase (Invitrogen, Dorset, UK) according to the manufacturers instructions [31]. Initially 200 μg (10 μl) total RNA was pre-incubated with 0.5 μg (1 μl) oligo-dT_12–18 _(Invitrogen, Paisley, UK) and 10 mM (1 μl) dNTP mix (Invitrogen, Paisley, UK) at 65° for 5 minutes. After chilling on ice, 4 μl of 5 × first strand buffer (containing 250 mM Tris-HCI (pH 8.3), 375 mM KC1, 15 mM MgCl_2_), 2 μl of 0.1 M DTT and 40 units (1 μl) of RNAse (Promega, Southhampton, UK) were added to each sample and the samples incubated for 2 minutes at 42°C, followed by the addition of 200 units (1 μl) of Superscript II reverse transcriptase (Invitrogen, Doreset, UK) and incubated for 50 minutes. Reverse transcriptase activity was terminated by incubation at 70°C for 15 minutes, and samples stored at -80°C until use.

### Real-time reverse transcriptase quantitative PCR assay design

Transcript sequences were obtained from the National Centre for Biotechnology Information [29] and were cross referenced to the Ensembl canine genome database [32]. Primer and probe sequences were then designed for each of the reference genes by using the Universal Probe Library Assay Design Centre (Roche Diagnostics Ltd; [33]) BLAST searches were performed for all primer sequences to confirm gene specificity, and electrophoresis of the PCR reaction mixture confirmed a single product of the appropriate length in all cases. Primers were synthesized by Metabion International AG (Martinsried, Germany), and probes were synthesized by Roche Diagnostics (Lewes, UK) using locked nucleic acid with 5'-end reporter dye fluorescein (FAM (6-carboxy fluorescene)) and 3'-end dark quencher dye.

Real-time RT-qPCR assays were performed in triplicate using the LightCycler^® ^480 (Roche Diagnostics; Lewes, UK) in 384 well format, with three no template controls used for each assay. The reaction volume in each well consisted of 5 μL LightCycler^® ^480 Probes Master 2 × concentration (Roche Diagnostics) (containing FastStart Taq DNA Polymerase, reaction buffer, dNTP mix (with dUTP) and 6.4 mM MgCl_2_), 0.7 μL of LightCycler^® ^480 Probes Master H_2_O (Roche Diagnostics), 0.1 μL of 20 μM forward primer, 0.1 μL of 20 μM reverse primer, 0.1 μL of 10 μM fluorescently-labelled probe and either 4 μL of sample cDNA, diluted template, or 4 μL of LightCycler^® ^480 Probes Master H_2_O. The standard amplification conditions consisted of 1 cycle at 95°C for 5 minutes, followed by 50 cycles of 95°C for 15 seconds and 60°C for 30 seconds. Real-time RT-qPCR data was then analysed by using LightCycler^® ^480 Basic Software (Roche Diagnostics; Lewes, UK). Standard curves were generated for each reference gene by employing cDNA or template oligonucleotides [34], the parameters of which are listed in Table 1. All samples were checked for absence of genomic DNA contamination using a canine genome specific RT-qPCR assay, previously described [25]. The assays were deemed to be reproducible, as determined by the average standard deviation of the triplicate repeats of each assay being less than 30% (Table 1).

### Reference gene stability analysis

Real-time RT-qPCR data was exported into an Excel datasheet (Microsoft Excel 2003) and analysed using three separate reference gene stability analysis software packages; geNorm [6],  *Bestkeeper©*[9] and NormFinder [11]. Each of these methods generates a measure of reference gene stability, which can be used to rank the reference genes in order of stability. GeNorm generates a stability measure (the M value) for each gene which is arbitrarily suggested to be lower than 0.4 (with a lower value indicating increased gene stability across samples), and a pairwise stability measure to determine the benefit of adding extra reference genes for the normalisation process, with again a lower value indicating greater stability of the normalised genes, and a lower value indicating greater stability with an arbitrary cut off value of lower than 0.15 indicating acceptable stability of the reference gene combination [6]. NormFinder generates a stability measure of which a lower value indicates increased stability in gene expression. By using a model-based approach, NormFinder groups samples to allow for a direct estimation of expression variation, compared to the pairwise comparison approach that ranks genes according to the similarity of their expression profiles. Therefore, taking a sample set which consists of two sample subgroups where all of the candidates but one show little difference between the groups, the one candidate which shows no difference will have the smallest stability value across all candidates and be the most stably expressed gene. *Bestkeeper©* generates a pairwise correlation co-efficient between each gene and the *Bestkeeper©* index (the geometric mean of the threshold cycle values of all the reference genes grouped together). Stability measures for combined (normal and diseased) samples were recorded, as ultimately it is these measures which would be used to determine which genes were suitable for normalising expression data from genes of interest in a particular disease (osteoarthritis) in practice.

*Bestkeeper©* can only be used to analyse a maximum of 10 housekeeping genes so the three genes least stably expressed (as determined by NormFinder) were excluded from *Bestkeeper©* analysis. The stability values for each gene, as determined by each method of analysis, are illustrated in Figure 2A, B, and 2C. Statistical tests were performed using a statistical software package (Minitab V14.1; Minitab Ltd.; Coventry, UK). Spearman rank correlation coefficients were then calculated using the ranking order of genes to compare the relationship of the relative ordering of genes by different methods of analysis (Table 2). Finally, the stability parameters of the new reference genes were compared to those generated for commonly used reference genes in a similar study of canine OA tissues [17] (Table 3).

## Authors' contributions

DNC and LM carried out the microarray data analysis. LM and FS carried out the assay design. DNC, LM and FS performed the molecular genetic studies and DNC performed the statistical analysis. DNC and PJRD conceived the study, its design and coordination, and drafted the manuscript with LM.

## References

[B1] Bustin SA, Nolan T (2004). Pitfalls of Quantitative Real-Time Reverse-Transcription Polymerase Chain Reaction. J Biomol Tech.

[B2] Imbeaud S, Graudens E, Boulanger V, Barlet X, Zaborski P, Eveno E, Mueller O, Schroeder A, Auffray C (2005). Towards standardization of RNA quality assessment using user-independent classifiers of microcapillary electrophoresis traces. Nucleic Acids Res.

[B3] Bustin SA (2002). Quantification of mRNA using real-time reverse transcription PCR (RT-PCR): trends and problems. J Mol Endocrinol.

[B4] Lekanne Deprez RH, Fijnvandraat AC, Ruijter JM, Moorman AFM (2002). Sensitivity and accuracy of quantitative real-time polymerase chain reaction using SYBR green I depends on cDNA synthesis conditions. Anal Biochem.

[B5] Huggett J, Dheda K, Bustin S, Zumla A (2005). Real-time RT-PCR normalisation; strategies and considerations. Genes Immun.

[B6] Vandesompele J, De Preter K, Pattyn F, Poppe B, Van Roy N, De Paepe A, Speleman F (2002). Accurate normalization of real-time quantitative RT-PCR data by geometric averaging of multiple internal control genes. Genome Biology.

[B7] Dheda K, Huggett JF, Bustin SA, Johnson MA, Rook G, Zumla A (2004). Validation of housekeeping genes for normalizing RNA expression in real-time PCR. Biotechniques.

[B8] Akilesh S, Shaffer DJ, Roopenian D (2003). Customized Molecular Phenotyping by Quantitative Gene Expression and Pattern Recognition Analysis. Genome Res.

[B9] Pfaffl MW, Tichopad A, Prgomet C, Neuvians TP (2004). Determination of stable housekeeping genes, differentially regulated target genes and sample integrity: BestKeeper--Excel-based tool using pair-wise correlations. Biotechnology Letters.

[B10] Haller F, Kulle B, Schwager S, Gunawan B, von Heydebreck A, Sultmann H, Fuzesi L (2004). Equivalence test in quantitative reverse transcription polymerase chain reaction: confirmation of reference genes suitable for normalization. Anal Biochem.

[B11] Andersen CL, Jensen JL, Orntoft TF (2004). Normalization of Real-Time Quantitative Reverse Transcription-PCR Data: A Model-Based Variance Estimation Approach to Identify Genes Suited for Normalization, Applied to Bladder and Colon Cancer Data Sets. Cancer Res.

[B12] Radonic A, Thulke S, Bae HG, Muller MA, Siegert W, Nitsche A (2005). Reference gene selection for quantitative real-time PCR analysis in virus infected cells: SARS corona virus, Yellow fever virus, Human Herpesvirus-6, Camelpox virus and Cytomegalovirus infections. Virology Journal.

[B13] Spinsanti G, Panti C, Lazzeri E, Marsili L, Casini S, Frati F, Fossi C (2006). Selection of reference genes for quantitative RT-PCR studies in striped dolphin (Stenella coeruleoalba) skin biopsies. BMC Molecular Biology.

[B14] de Brouwer AP, van Bokhoven H, Kremer H (2006). Comparison of 12 reference genes for normalization of gene expression levels in Epstein-Barr virus-transformed lymphoblastoid cell lines and fibroblasts. Molecular Diagnosis and Therapeutics.

[B15] Saviozzi S, Cordero F, Lo M, Novello S, Giorgio VS, Calogero R (2006). Selection of suitable reference genes for accurate normalization of gene expression profile studies in non-small cell lung cancer. BMC Cancer.

[B16] Szabo A, Perou CM, Karaca M, Perreard L, Quackenbush JF, Bernard PS (2004). Statistical modeling for selecting housekeeper genes. Genome Biology.

[B17] Ayers D, Clements DN, Salway F, Day PJR (2006). Expression stability of commonly used Control Genes in Canine Articular Connective Tissues. Submitted for publication.

[B18] Gorzelniak K, Janke J, Engeli S, Sharma AM (2001). Validation of endogenous controls for gene expression studies in human adipocytes and preadipocytes. Horm Metab Res.

[B19] Matyas JR, Huang D, Adams ME (1999). A comparison of various "housekeeping" probes for northern analysis of normal and osteoarthritic articular cartilage RNA. Connect Tissue Res.

[B20] Bogaert L, Van Poucke M, De Baere C, Peelman L, Gasthuys F, Martens A (2006). Selection of a set of reliable reference genes for quantitative real-time PCR in normal equine skin and in equine sarcoids. BMC Biotechnology.

[B21] Brinkhof B, Spee B, Rothuizen J, Penning LC (2006). Development and evaluation of canine reference genes for accurate quantification of gene expression. Anal Biochem.

[B22] Cavdar Koc E, Blackburn K, Burkhart W, Spremulli LL (1999). Identification of a Mammalian Mitochondrial Homolog of Ribosomal Protein S7. Biochem Biophys Res Commun.

[B23] Listgarten J, Graham K, Damaraju S, Cass C, Mackey J, Zanke B (2003). Clinically validated benchmarking of normalisation techniques for two-colour oligonucleotide spotted microarray slides. Applied Bioinformatics.

[B24] Ayers D, Clements D, Salway F, Day P (2007). Expression stability of commonly used reference genes in canine articular connective tissues. BMC Veterinary Research.

[B25] Clements DN, Carter SD, Innes JF, Ollier WE, Day PJ (2006). Analysis of normal and osteoarthritic canine cartilage mRNA expression by quantitative-PCR. Arthritis Res Ther.

[B26] Yu H, Luscombe NM, Qian J, Gerstein M (2003). Genomic analysis of gene expression relationships in transcriptional regulatory networks. Trends Genet.

[B27] Maccoux LJ, Salway F, Day PJR, Clements DN (2007). Expression profiling of select cytokines in canine osteoarthritis tissues. Vet Immunol Immunopathol.

[B28] Jones P, Jones C, Fretwell N, Martin A, Soloviev M (2004). Design and production of a whole genome dog oligonucleotide microarray. Advances in Canine and Feline Genomics.

[B29] Information NCB (2007). National Centre for Biotechnology Information. http://www.ncbi.nlm.nih.gov.

[B30] Clements DN, Vaughan-Thomas A, Peansukmanee S, Carter SD, Innes JF, Ollier WER, Clegg PD (2006). Assessment of the use of RNA quality metrics for the screening of normal and pathological canine articular cartilage samples. Am J Vet Res.

[B31] Invitrogen (2006). Invitrogen. http://www.invitrogen.com.

[B32] Ensembl (2007). Ensembl. http://www.ensembl.org.

[B33] Ltd RD (2007). Roche Diagnostics Ltd. http://www.roche-applied-science.com.

[B34] Mohammadi M, Day PJR (2004). Oligonucleotides used as template calibrators for general application in quantitative polymerase chain reaction. Anal Biochem.

